# Testing the Coding Potential of Conserved Short Genomic Sequences

**DOI:** 10.1155/2010/287070

**Published:** 2010-03-08

**Authors:** Jing Wu

**Affiliations:** Department of Statistics, Carnegie Mellon University, PA 15213, USA

## Abstract

Proposed is a procedure to test whether a genomic sequence contains coding DNA, called a *coding potential region*. The procedure tests the coding potential of conserved short genomic sequence, in which the assumptions on the probability models of gene structures are relaxed. Thus, it is expected to provide additional candidate regions that contain coding DNAs to the current genomic database. The procedure was applied to the set of highly conserved human-mouse sequences in the genome database at the University of California at Santa Cruz. For sequences containing RefSeq coding exons, the procedure detected 91.3% regions having coding potential in this set, which covers 83% of the human RefSeq coding exons, at a 2.6% false positive rate. The procedure detected 12,688 novel short regions with coding potential at the false discovery rate <0.05; 65.7% of the novel regions are between annotated genes.

## 1. Introduction

 A popular computational strategy in identifying coding DNA of the human genome is using probability models. For example, for a single genome, one approach would be to use probability models to delineate a DNA sequence into a gene which is composed of several parts such as promoter regions, UTR regions, splicing sites, exons, and so forth [1]. Alternatively, by considering a genome (e.g., human) together with the genome of a suitably related species (e.g., mouse), one can combine the conserved information of the two species to develop a more refined probability models for the gene portions (ROSETTA [2], CEM [3], TWINSCAN [4], SLAM [5], and SGP2 [6–8]). While these approaches have been effective in predicting genes, a noticeable drawback is that the more refined a probability model is, the more constraints there are for a DNA sequence to be a gene. In effect, a highly refined probability model tends to overparameterize the problem, and thus inevitably restrain the ability of a gene prediction algorithm for identifying genes, especially those that do not fit well with the “prescribed characters” delineated by the probability model; see for example [9]. To compensate such restraint, some algorithms report genes that are not the best fit to the model (e.g., suboptimal genes in GENSCAN).

Noting the limitations of existing approaches motivated our interest to identify *coding potential regions*. That is, to localize regions that contain coding DNA, we develop procedures that determine the coding potential of short regions. Instead of slightly relaxing the restraints on gene structure, such as in the prediction of suboptimal genes in GENSCAN, the proposed method tries to make probabilistic assumptions on gene structure as few as possible. The approach employs a locally smooth function, that is, the lowess function [10]. The key idea is that the signal contained in each codon is generally faint and not strong enough to stand out from the background noise, but fortunately each coding exon in the gene is made of a block of codons, so that by using a locally smooth function one is able to collect the strength of such faint signals from codons together to determine the coding potential of the region. The proposed procedure is mainly based on probability models for the nucleotide dependency in codons and the dependency of nucleotide triplets across different sequences. A log-odds ratio is calculated for each triplet in the human genome, to measure the likelihood of the triplet being random or a codon [7, 11–13]. The intuition is that when there is a coding exon in the aligned sequences, there is the associated peak in the log-odds ratio. Therefore, the coding potential of a region can be viewed as the presence of a peak in the sequence of log-odds ratios, under the expectation that a locally smooth function may be useful. The difference between the proposed method and the existing gene prediction method is that it tries to tell whether a sequence contains a coding region or not instead of trying to obtain the boundary of a coding exon in the sequence. The nonparametric nature of such an approach is expected to provide regions in genes with novel structure.

## 2. Method

**Figure 1:**
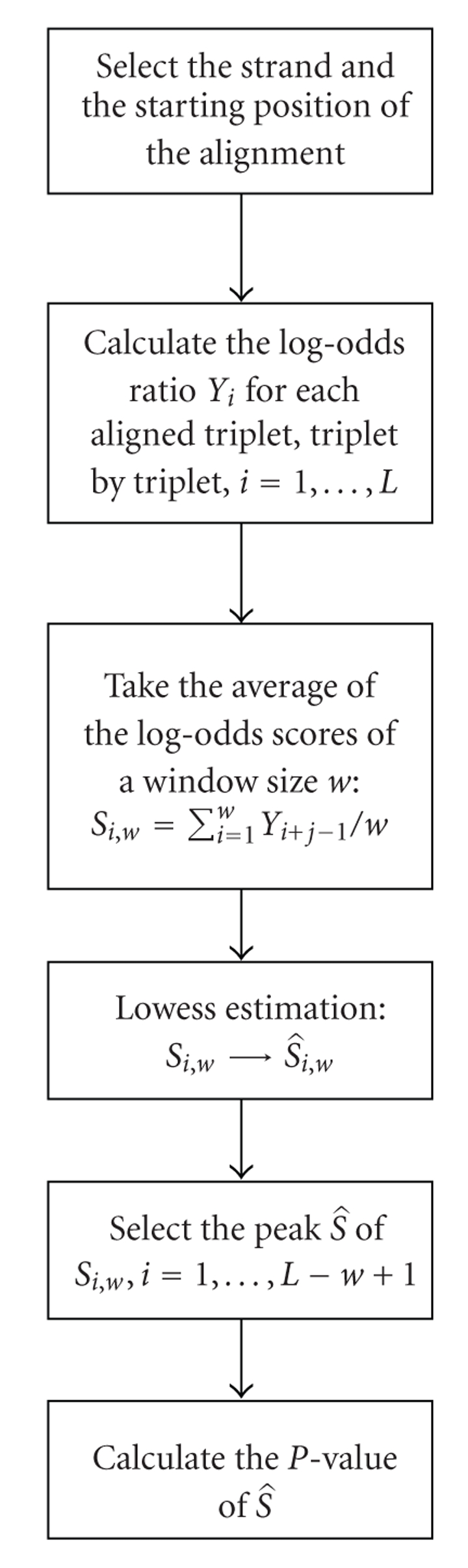
Summary of the proposed statistical procedure.

The proposed procedure is detailed schematically in Figure 1. First, given the likelihood of an aligned triplet pair from a codon, the aligned sequence pair is segmented into aligned triplet pairs and transformed into log-odds ratios. Second, a window frame with a given size slides through the series of log-odds ratios and the average log-odds ratio in each window frame is obtained. Third, the average log-odds ratio is smoothed by a locally smooth method [10], that is, the lowess method, which is a robust locally weighted regression. Finally, the largest local maximum of the corresponding lowess function is selected as the test statistic and the approximate *p*-value of the test statistic is proposed. The proposed method brings statistical tools such as the locally smooth function to the coding potential detection problem. It treats the coding potential problem as a peak hunting problem. The proposed method not only realizes the optimal accuracy suggested by [12], but also detects novel regions with high coding potential.

### 2.1. Hypotheses

The proposed procedure is based on the observations that functional elements, such as the codons of exons, tend to be more strongly conserved in evolution than random genomic sequences and that adjacent codons tend to depend on each other. The method is applicable to data that consists of genomic sequences of interest, called the *target sequence*, and sequences from a related species that are aligned to the target sequence, called the *information sequence*. The test of the alignment discriminates between the following hypotheses:

(*H*_0_) all the DNA in the target sequence is not coding,
(*H*_1_) a proportion of the DNA in the target sequence is coding.

Thus, a region has coding potential when (*H*_0_) is rejected.

### 2.2. Model

The approach to determine a region's coding potential is to use information provided by the log-odds ratio of the aligned triplet pairs in the given alignment. The log-odds ratio is defined as follows. Denote a pair of aligned sequences *X* = {*h*_1_,…, *h*_*L*_; *m*_1_,…, *m*_*L*_}, where *h*_*i*_'s are non-overlapping triplets in the target sequence and *m*_*i*_ is the triplet in the information sequence aligned to *h*_*i*_. The log-odds ratio (LOD) at each position *i*, *i* = 2,…, *L*, is

(1) $$\begin{array}{c} {\text{LOD}}_{i}=\log\;\frac{P_{A}\left(h_{i}\;|\;h_{i-1}\right)P_{B}\left(m_{i}\;|\;h_{i}\right)}{Q_{A}\left(h_{i}\;|\;h_{i-1}\right)Q_{B}\left(m_{i}\;|\;h_{i}\right)}, \end{array}$$

where probability matrix *P*_*A*_ gives the conditional probability of observing codon *h*_*i*_ given the previous codon *h*_*i*−1_, *P*_*B*_ gives the conditional probability of observing an aligned triplet *m*_*i*_ given codon *h*_*i*_, *Q*_*A*_ gives the conditional probability of observing a triplet *h*_*i*_ from noncoding regions given the previous triplet *h*_*i*−1_, and *Q*_*B*_ gives the conditional probability of observing an aligned triplet *m*_*i*_ given *h*_*i*_ from noncoding regions.

The concept of constructing a test statistic that identifies an exon based on the log-odds score is that for a target sequence containing an exon; when the partitioning of the alignment into aligned triplets is correct, there is a position *l*_0_ and a position *l*_1_ such that *h*_*l*_0__,…, *h*_*l*_1__ are codons while *h*_1_,…, *h*_*l*_0_−1_ and *h*_*l*_1_+1_,…, *h*_*L*_ are not codons. Therefore, *l*_0_ and *l*_1_ are the two points where the underlying distribution of *X*_*j*_ = (*h*_*j*_, *m*_*j*_) switches between that of the random triplet-triplet alignment and the codon-triplet alignment, thus resulting in the log-odds ratios between *l*_0_ and *l*_1_ with a higher mean. When using a nonparametric method to smooth the log-odds ratios, the corresponding curve of the smoothed log-odds score versus its location in the alignment will show a peak between *l*_0_ and *l*_1_.

To obtain the value of the test statistic from a given alignment, the first step is to partition the alignment into aligned triplets so that the codons are in the correct frame and the correct DNA strand when the alignment contains a coding exon. To obtain the segmentation, the average log-odds ratio, *S*_*i*,*w*_0__ = ∑_*j*=1_^*w*_0_^LOD_*i*+*j*−1_/*w*_0_, is calculated for each block of *w*_0_ aligned triplet pairs for both the alignment and the reverse complement of the alignment. The block that attains the maximum *S*_*i*,*w*_0__ is extended toward both ends of the alignment in units of aligned triplet pairs. Removing any partial triplet pairs at the both ends of the alignment, the segmentation and the strand of the alignment is obtained and denoted by *X* = {*h*_1_,…, *h*_*L*_; *m*_1_,…, *m*_*L*_}.

Given the selected segmentation and strand, *X* = {*h*_1_,…, *h*_*L*_; *m*_1_,…, *m*_*L*_}, the average log-odds scores, *S*_*i*,*w*_ = ∑_*j*=1_^*w*^LOD_*i*+*j*−1_/*w*, are obtained for the *i*th aligned triplet pair, where LOD_*k*_ is defined in (1) and *w* is a parameter. Because the nucleotides in the noncoding region are less conserved in evolution, the nucleotides in noncoding regions are assumed to be independent, so *S*_*i*,*w*_ is approximately normally distributed when *w* is large enough.

The function * lowess()* in the *R* standard package (http://www.r-project.org/) is used to smooth the average log-odds scores. A smoothing parameter *f* determines the fraction of neighboring data points to be used in smoothing. Since longer exons tend to have longer alignments, *f* is fixed for all alignments so that the length of the exon is taken into account. Based on this smoothing, detecting the exon in the alignment is transformed into detecting a significant peak in the profile of the smoothed average log-odds scores.

The maximum of the local maximum, denoted by $\hat{S}$, of the lowess estimation is selected as the test statistic. The selection of the local maximum is performed by the function * ppc.peaks()* in the * R* package * ppc* developed by Tibshirani et al. [14], in which the parameter span is set as the same as *f* in the lowess function.

Finally, the *p*-value of the test statistic $\hat{S}$ is approximated by the extreme distribution of the normal random variable. Specifically, since the scores *S*_*i*,*w*_'s are normally distributed, the lowess smoothed scores, denoted by ${\hat{S}}_{i,w}$ are also normally distributed [10]. Moreover, since *S*_*i*,*w*_'s are locally dependent, for simplicity, they are treated as if they were independent under the null hypothesis. Denoting *P*_0_ as the probability that a peak exists in the alignment and assuming that ${\hat{S}}_{i,w}$ is from a normal distribution, by the Bayesian rule, the approximate *p*-value for $\hat{S}$ is

(2) $$\begin{array}{cc} p & =P(\max\;\left({\hat{S}}_{1,w},\ldots ,{\hat{S}}_{L-w+1,w}\right)>\hat{S}\;|\;\text{peak}\;\text{exists}\;\text{under}\;H_{0})P_{0}\\ & \approx \left({1-P\left(Z<\frac{\hat{S}-\mu }{\sigma }\right)}^{L-w+1}\right)P_{0}, \end{array}$$

where *Z* ~ *N*(0,1). The *p*-value is set as *p* = 1 when no peak is found. Given a significance level *α*, when the *p*-value of an alignment is less than *α*, the alternative that the alignment contains coding DNA is supported. When testing *k* alignments, the  *p*-values, *p*_1_,…, *p*_*k*_, are transformed into *q*-values to control the false discovery rate [15, 16], where the false discovery rate is the proportion of false rejections of *H*_0_ among the total number of rejections of *H*_0_. That is, denote *r*_*i*_ as the rank of *p*_*i*_ with the smallest *p*-value ranked as 1 and let

(3) $$\begin{array}{c} q_{i}=\min\;\left(\frac{kp_{i}}{r_{i}},1\right), \end{array}$$

then the expected number of false positive is ≤ *r*_*i*_0__ *α*, where *r*_*i*_0__  =  max {*r*_*i*_ : *q*_*i*_ < *α*}.

### 2.3. Datasets

The proposed method is assessed on the set of highly conserved human-mouse pairwise alignments, that is, the *axtTight* directory of the UCSC genome database in Human May 2004 (hg17) (http://hgdownload.cse.ucsc.edu/goldenPath/hg17/vsMm5/axtTight/). This *axtTight* folder contains the latest version of a highly conserved subset of the best alignments with mouse sequences for any part of the human genome; it remains the same although the genome database has been updated to hg19. The alignments are quite short; about 95% of the human sequences in this set are <597 bps. An interesting feature of this set is that, although it was obtained without the knowledge of gene structure, it contains a subset that heavily overlaps with the set of human RefSeq coding exons [17, 18] (http://www.ncbi.nih.gov/RefSeq/) in the genome database at UCSC (http://genome.ucsc.edu/cgi-bin/hgTables), May 2004, which has 172,042 exons nonoverlapping with each other. The human sequences in the *axtTight* folder overlap with 91.2% human RefSeq coding exons, in which 94.8% sequences overlap with only one RefSeq coding exon in each sequence, 4.0% overlap with only two RefSeq coding exons, and the average percentage of coding DNA in the human sequences that overlap with human RefSeq coding exons is 67%. Thus, the human sequences in this folder were used for both evaluating the procedure and for determining novel regions with coding potential. To be consistent with the coordinates of the sequences in the *axtTight* folder, the parameters for the proposed method were estimated from the sequences in the assembly of hg17.

Since the proposed method tests whether coding DNAs are embedded in the target sequence, the positive set consists of alignments whose target sequence contains a coding exon with noncoding DNA flanking it. The negative set consists of alignments whose target sequence does not have evidence of coding DNA.

In order to determine regions with coding potential in the *axtTight* folder, the human sequences were extracted from the alignments in the *axtTight* folder, and each sequence was extended 50 bps on each end and paired with the mouse sequence according to the alignments in the *axtNet* folder (http://hgdownload.cse.ucsc.edu/goldenPath/hg17/vsMm5/axtNet/). The alignments that are longer than 150 bps were kept. The human sequences of the alignments (before extension) overlapping with RefSeq coding exons, are called the *conserved coding potential regions*. Among these alignments, 3,000 were randomly selected as a training set. The human sequences in the *axtTight* folder, whose extended alignments are longer than 150 bps, but do not overlap with the human RefSeq coding exons are called *candidate coding potential regions*. The total number of conserved coding potential region is 146,254, which corresponds to 3.9 × 10^7^ bps and includes 156,928 RefSeq coding exons. The average percentage of coding DNA in the human sequence of the extended alignment of the conserved coding potential region is 43%. The total number of candidate coding potential regions is 751,313, corresponding to 1.2 × 10^8^ bps. To show the robustness of the proposed method, the human-dog alignments of the extended conserved coding potential regions were also extracted from hg17. In this set, the average percentage of coding DNA in the human sequence of the extended alignment of the conserved coding potential region is 38% since more noncoding flanking DNAs are conserved between human and dog.

To simulate aligned conserved noncoding regions, we first estimated the conditional probability of the adjacent nucleotide triplet pair in human, the aligned nucleotide triplet pair between human and mouse, and the length distribution of conserved noncoding regions from the set of aligned human-mouse sequences called the alignment of potential nonexons [7]. These sequences do not overlap with any known genes, ESTs. The coordinates of the potential nonexons from [7] were lifted from hg12 to the assembly of hg17 in UCSC's genome database by the batch coordinate conversion (http://genome.ucsc.edu/cgi-bin/hgLiftOver). The alignments of potential nonexons were then extracted from the *axtNet* folder in UCSC's genome database (hg17) and 20,000 alignments were randomly selected as a training set. Based on the estimated probabilities and the length distribution from the training set for the alignment of noncoding regions, 15,062 paired sequences were simulated. Among them, 10,305 paired sequences are longer than 150 bps and are used as noncoding regions to evaluate the proposed procedure.

Finally, to analyze the coding potential regions detected from the *axtTight* folder, the predictions of existing gene and pseudogene prediction algorithms listed in Table 1 from the genes and gene prediction tracks in UCSC's genome database (http://genome.ucsc.edu/cgi-bin/hgTables, human, May 2004) were downloaded.

### 2.4. Training the Model

In order to apply the testing procedure, the probabilities under the codon model and the noncoding region model in (1) were estimated. The conditional probability of two triplets is estimated by the joint counts from the alignments in the training sets. That is,

(4) $$\begin{array}{cc} P_{A}\left(h\;|\;h^{\prime }\right) & =\frac{\text{Number}\;\text{of}\;\text{pairs}\;\;\left(h^{\prime }h\right)+e}{\text{Number}\;\text{of}\;h^{\prime }+125e},\\ P_{B}\left(m\;|\;h\right) & =\frac{\text{Number}\;\text{of}\;\text{pairs}\;\;\left(hm\right)+e}{\text{Number}\;\text{of}\;h+125e},\\ Q_{A}\left(a\;|\;a^{\prime }\right) & =\frac{\text{Number}\;\text{of}\;\text{pairs}\;\;\left(a^{\prime }a\right)+e}{\text{Number}\;\text{of}\;a^{\prime }+125e},\\ Q_{B}\left(b\;|\;a\right) & =\frac{\text{Number}\;\text{of}\;\text{pairs}\;\;\left(ab\right)+e}{\text{Number}\;\text{of}\;a+125e}, \end{array}$$

where *e* = 1 is the pseudocount added, *h* and *h*′ are adjacent codons in conserved coding regions, *m* is the triplet aligned to *h*, *a* and *a*′ are adjacent triplets in potential nonexons, and *b* is the triplet aligned to *a*. Each probability matrix is of dimension 125 × 125. The probability matrices can be downloaded from http://www.stat.cmu.edu/~jwu/axtTight/probs/. For any two nucleotide triplets *c*_1_ *c*_2_ *c*_3_ and *d*_1_ *d*_2_ *d*_3_, *c*_*k*_, *d*_*k*_∈{*A*, *C*, *G*, *T*, indel}, the nucleotides are coded as *A* = 0, *T* = 1, *G* = 2, *C* = 3, indel = 4, *P*(*d*_1_ *d*_2_ *d*_3_ ∣ *c*_1_ *c*_2_ *c*_3_) corresponding to the (*i*, *j*)th entry *i* = 25*c*_1_ + 5*c*_2_ + *c*_3_, *j* = 25*d*_1_ + 5*d*_2_ + *d*_3_, so each probability matrix is of dimension 125 × 125.

**Figure 2:**
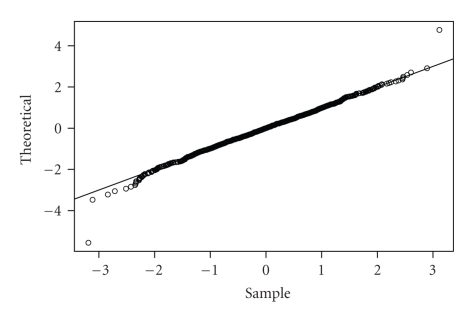
A normal qq-plot of the averaged log-odds scores from the simulated sequences.

The window sizes are set at *w*_0_ = 20 and *w* = 9 which correspond, respectively, to the 10th and the 2nd percentile of the length distribution (in units of triplets) of the exons in the training set. The normal qq-plot in Figure 2 illustrated the distribution of the score *S*_*i*,*w*_ as normal, which is consistent with the assumption for the *p*-value calculation. 

The estimated mean and variance of the log-odds scores for the simulated triplets are −0.66 and 1.58, respectively. Since *w* = 9, the estimated parameters in (2) are $\hat{\mu }=-0.66$ and $\hat{\sigma }=1.58/3=0.527$. For each alignment in the test sets, the *p*-value is $p\approx (1-P(Z<(\hat{S}+0.66)/0.527{)}^{L-8})\times P_{0}$, where *Z* is from standard Normal *N*(0,1) and *L* is the number of log-odds scores.

Lastly, the parameter *f* in *lowess()* and span = *f* in *ppc.peaks()* are selected by testing the alignment in the training set of conserved coding regions and potential noncoding DNAs. An appropriate *f* uses as many of the neighboring scores as possible to smooth the averaged log-odds score in the center of the exon in the coding region but includes few scores from noncoding DNAs. Since in the extended alignment of conserved coding regions, on average, each alignment contains 43% coding DNAs, only *f* ≤ 0.5 were considered. To select *f*, values 1/4, 1/3, and 1/2 were evaluated on the training datasets. For each *f*, *P*_0_ is estimated by the observed relative frequency of the potential nonexon alignments having a peak and then the *p*-value in (2) is obtained for each alignment. Among them, the *p*-values from *f* = 1/3 best separate the extended alignments in the training set of conserved coding regions from potential nonexons. Thus, the parameter *f* in * lowess()* is set as *f* = 1/3 and then the estimated probability of observing at least one peak in noncoding regions is *P*_0_ = 0.04. For each alignment in the test sets, the *p*-value is $p\approx (1-P(Z<(\hat{S}+0.66)/0.527{)}^{L-8})\times 0.04$, where *Z* is from *N*(0,1) and *L* is the number of log-odds scores.

## 3. Results

The procedure is tested on the human sequences in the *axtTight* folder in UCSC's genome database (http://hgdownload.cse.ucsc.edu/goldenPath/hg17/vsMm5/axtTight/). From this set, the procedure detected 91.1% conserved coding potential regions using human-mouse alignments, with the estimated 2.6% false positive rate, covering to 83% of the entire human RefSeq coding exons. At the same false positive rate, it also detects 90.7% conserved coding potential regions using human-dog alignments. Among the detected conserved coding potential regions from human-mouse alignments, many contain short coding exons and coding exons with alternative splicing sites which existing gene prediction algorithms tend to miss. In addition, the procedure identified 12,688 human sequences at the false discovery rate <0.05; among them, 57 overlap with nonhuman RefSeq coding exons [24], 65.7% are between annotated genes, and 41.4% have UniGene [29] matches, indicating that these regions may contain novel coding exons.

### 3.1. Detecting Coding Potential Regions from the Datasets

**Figure 3:**
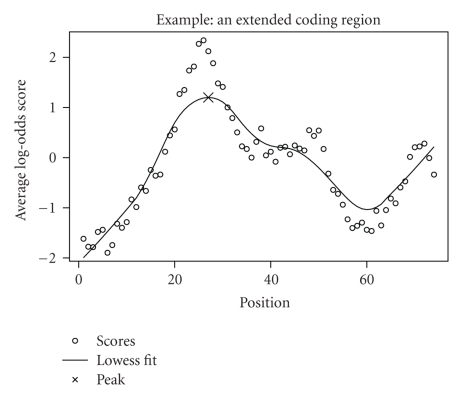
Identifying a coding potential region in chromosome 1: 1058121-1058365 from assembly hg17. The position is in units of triplets. The codons are at position 25–56.

Figure 3 illustrates an example of identifying a coding potential region of human chromosome 1: 1058121-1058365, in which 1058195-1058290 is a coding exon. 

The plot in Figure 3 shows the 74 averaged log-odds scores from a selected segmentation of the alignment of that conserved region. From the lowess fit and peak selection, as indicated by the solid curve and the cross patch, respectively, the value of the test statistic is obtained at the peak $\hat{S}=1.199$ having *P* = 0.0004.

The performance of the proposed procedure is compared with the results of Nekrutenko et al. (2002) [12]. Their study shows that, when an aligned sequence is either an aligned coding exon with codon frame known (true positive) or an aligned random sequence (true negative), the likelihood ratio test attains the true positive rate (TP) of 90.5% and the false positive rate (FP) of 2.6.% This result can be viewed as the best accuracy that coding potential region detection methods can attain using only conservation information since the true positive set assumes that the coding exon frames are known. Our negative set includes 10,305 simulated paired sequences that are at least 150 bps. This set is comparable to the number of simulated paired sequences used in [12], which is 24,000 without length limitation. To detect the coding potential region when the coding exon frames are unknown, the error rates of the proposed method are calculated as follows. Given a threshold, the true positive rate is the fraction of the total number of conserved coding potential regions whose alignment has *p* < *α* and the false positive rate is the fraction of the total number of simulated alignments having *p* < *α*. The results are summarized in Table 2.

To further study the coding potential regions detected in the test set, we compared the detection on RefSeq coding exons with GENSCAN and TWINSCAN with regards to the type of exons and summarized the results in Table 3. For single exons, because the gene structure is simple, GENSCAN can take the full advantage of the gene structure without the conservation limit; it is able to identify most single exons. Using sequence conservation limited the ability to identify unconserved genes as shown by the predictions from TWINSCAN and the proposed method.

We also compared the results with the internal exons predicted by MZEF [30] in Table 1 in [30]. We identified the locations of 22 genes in UCSC's genome database. Since the genomic region has expanded over the years, we compare the percentage of the internal exons identified relative to the number of internal exons available to both methods per gene. Among these genes, the proposed method had a higher call rate than MZEF on internal exons in 9 genes and had a lower call rate on those in another 10 genes. The average call rate for the proposed method on the 22 gene is 76% while that of MZEF is 83%. On the other hand, when only counting the regions available in the test set, the average call rate for the proposed method is 88.6%.

We examined the regions that are conserved noncoding regions defined by PhastCons [31]. PhastCons defined 39% of the sequences in the axtTight set as conserved noncoding regions, and in the subset of sequences with coding potential with *p* < 0.0387, only 22% are defined as conserved noncoding region. We also evaluated the structured RNAs in the ENCODE [32] regions, that is, Vienna RNAz [33]. We downloaded the encodeUViennaRnaz table from UCSC's genome database. Among the total 3,346 conserved RNA regions in the encodeUViennaRnaz table, our dataset * axtTight* overlaps with 489 regions and 251 of them have a *p*-value < 0.038. We also examined closely the regions that were not predicted by those computational algorithms in Table 1 and found that most of those regions contain coding exons of alternative splicing sites or very short coding exons. For example, the region chr1:198070085-198070137,…tagccaGAGCAGGAAGgacat…, contains one internal coding exon indicated by the upper case. The *p*-value is 0.007. It is not predicted by any of the algorithms mostly because this exon lacks the proper flanking dinucleotides (GT/AG or GC/AG). Another example is the region chr1:211644829-211645072; it only contains a coding exon which is the “A” of the start codon. The *p*-value is 0.002. This coding exon is only predicted by AceView which considers alternative splicing.

### 3.2. Detecting Novel Coding Potential Regions in the Human Genome

The proposed method is also applied to the alignments of candidate coding potential regions to detect novel coding potential regions. To adjust for multiple hypothesis testing, the *p*-value is adjusted to the *q*-value according to (3) to control the false discovery rate. By setting *q*< = 0.05, which corresponds to *p* < 0.01, we detected 46,188 coding potential regions. Among them, 12,688 are absent from the predictions listed in Table 1 (excluding nonhuman RefSeq genes and UniGene genes). Among the human segments containing novel coding exons, 57 overlap with nonhuman RefSeq coding exons [24] and 5,259 (41.4%) have UniGene matches. These evidences indicate the existence of 12,688 novel coding potential regions in human. The coordinates of the human segments of these regions can be downloaded from http://www.stat.cmu.edu/~jwu/axtTightCoding/.

The novel coding potential regions detected are compared with those by Nekrutenko et al. [13], in which they reported 13,700 novel coding exons; 61% of which lay within annotated genes and 38% lay between annotated genes, and among those between annotated genes, 25% had UniGene matches. Among the 12,688 novel coding potential regions reported here, 34.3% are within annotated genes and 65.7% are between annotated genes according to the annotation in Table 1, and among the novel coding potential regions in between annotated genes, 35.1% have UniGene matches. The difference shows that the proposed method is more sensitive to genes with unknown structure.

## 4. Discussion

 A statistical procedure is proposed to detect regions containing coding exons in conserved human sequences. It reveals coding potential regions from genes that do not fit the structure prescribed by existing methods. The success of the procedure depends on a locally smooth function (i.e., the lowess function) to address the problem of localizing coding potential regions. Furthermore, the prediction method is sensitive to codons but insensitive to noncoding DNA. As seen from the results from human-mouse alignments and human-dog alignments (Table 2), the method is also not sensitive to the alignments used.

The proposed method is an effective tool to analyze short conserved regions. Although it does not predict gene structures from sequences, it identifies those conserved regions that overlap with genes. A direct application of the proposed method is to improve the accuracy of the existing gene or coding exon prediction algorithms. The proposed method could be used as a filtering procedure to provide input sequences to these exon prediction algorithms. For example, when applied to the data in short HMM [7], with the same parameters except that the probability matrices (4) were estimated from the training data in [7], it reduced false positive from 0.77% to 0.49% at same true positive rate by filtering out the alignments with large *p*-values. It could also be used as an additional criterion for the alternative genes predicted by GENSCAN. In addition, the proposed method would also benefit algorithms that predict single-exon genes. Specifically, by increasing the window size *w* and applying to the sets with longer flanking noncoding regions, the peak in the hump in the long coding exon emerges while the peak in the humps in other short exons becomes less significant. Then, using the detected coding potential regions as the input data for algorithms that only predict the single-exon gene, because of the gene structure, one would expect that most long exons from multiexon genes would be filtered out.

A more interesting feature of the proposed method is that it provides new data for methods that predict gene structures. As shown in Section 3.1, from the comparison with GENSCAN, the proposed method detects more coding potential regions from multiple-exon genes. Moreover, it is sensitive to coding potential regions containing short exons and exons with alternative splicing sites as shown in Section 3.1. Thus, the proposed method could be used to reveal novel gene structure by studying the coding potential regions that failed to be predicted by the existing algorithms.

There is a possibility that the proposed method could be biased toward pseudogenes simply because there is a relaxation of the whole gene structure. However, such a bias is not obvious since the percentage of coding potential regions predicted overlapping with known pseudogenes is within the range of those from existing gene prediction algorithms. As a matter of fact, 2% of the coding potential regions predicted from the human sequences in the *axtTight* folder overlap with the database of Yale pseudogenes (http://www.pseudogene.org/), corresponding to 4% in length. Both percentages are lower than those of GENEID, GENSCAN, Augustus, and SGP and are higher than those of the rest 7 gene prediction methods in Table 1 (excluding RefSeq genes, nonhuman RefSeq genes, vega genes, vega pseudogenes, retro genes, and Yale pseudogenes).

The proposed statistical procedure is not sensitive to the parameters used since the lowess function smoothes out the sudden changes in the log-odds scores from the randomness. However, there still are some general rules for selecting the parameters. Specifically, the window size *w*_0_ for selecting the strand and segmentation of the alignment should be large enough to include more codons, but not too large so that few noncoding DNAs are included when the window frame is on the coding exon. The window size *w* for obtaining the normally distributed scores should be small so that the dependency among the scores is weak and the alignment has ample scores for the lowess estimation and the peak selection. On the other hand, *w* should also be large enough to ensure the distribution of the average log-odds ratios in the window frame is approximately normal. The method is not sensitive to the parameter *f* in the lowess function or the parameter *span* in *ppc.peaks()* due to the nonparametric nature of these two functions. Moreover, the lowess function could be replaced by similar locally smooth functions such as the spline method; other peak selection functions could also be used instead of *ppc.peaks()*. However, the smoothing parameter does affect the prediction sensitivity. The larger the *f*, the larger the *p*-value for a given alignment. On the other hand, as shown in Table 2, for a dataset that is not dramatically different from the one used in this paper in DNA composition and sequence length distribution, the threshold for the *p*-value; say 0.01, remains a good indication on whether the sequence contains coding DNA or not.

One limitation of the proposed method is that it is only applicable to alignments that are not too short; say longer than 150 bps. This limitation excluded 3.5% of human RefSeq coding exons that overlap with the alignments in the *axtTight* folder from the analysis, as these RefSeq coding exons do not have enough conserved flanking noncoding regions after the extension. One justification of the length constraint is to insure that the alignment has adequate log-odds scores for the peak selection function * ppc.peaks()*. Furthermore, the proposed method is expected to have limited statistical power in detecting coding potential regions from alignments ≤150 bps. As shown by Nekrutenko et al. [12], even with gene structure given, only 42% coding exons are detected from the conserved RefSeq coding exons with length ≤50 bps. The power of the proposed method on the short aligned sequences (<150 bps) is about 40%. Also, the power of proposed approach decreases when the length of the alignment increases to thousands of base pairs or more since the *p*-value increases with the length of the alignment.

The code that realizes the proposed procedure and the predicted coding potential regions can be downloaded from http://www.stat.cmu.edu/~jwu/axtTightCoding/, in which the code to calculate the log-odds score is written in C++ and the code to calculate the *p*-value is written in * R*.

## Figures and Tables

**Table 1:** The above tables in UCSC's genome database are used to analyze the coding potential regions detected from the human sequences in the *axtTight* folder in UCSC's genome database.

Tracks	URL
RefSeq [17, 18]	http://www.ncbi.nih.gov/Refseq/
Known genes [19]	
TWINSCAN [4]	
GENSCAN [1]	
SGP [20]	http://nemo.imim.es/grib/
ENSEMBL	http://www.ensembl.org/
GENEID [21]	http://www1.imim.es/software/geneid/index.html
AUGUSTUS [22]	
ECgene [23]	http://genome.ewha.ac.kr/ECgene/
MGC [24]	
AceView [25]	http://www.ncbi.nih.gov/IEB/Research/Acembly/index.html
CCDS [18, 26]	
Nonhuman RefSeq [24]	
Retropose [27]	
Yale Psuedo [28]	http://www.pseudogene.org/
Vega	http://vega.sanger.ac.uk/
Vega pseudogenes	http://vega.sanger.ac.uk/
UniGene [29]	

**Table 2:** The detection of coding potential regions in the human-mouse conserved regions. The table lists the number of alignments and the corresponding base pairs of the human sequences in each test set. The true positive rates and false positive rates correspond to the number of alignments that have *p*-value less than *α* = 0.0387 by the present method, where the method with the parameters estimated from human-mouse training sets was applied both to the human-mouse alignments and human-dog alignments. The row of *K*_*A*_/*K*_*S*_ is cited from [12]. The threshold is set so that the false positive rate of the proposed method is the same as that of [12].

	Conserved coding regions (TP)	Simulated random sequence pair (FP)
Size	146,254	10,305
	(3.9 × 10^7^ bps)	(6.8 × 10^6^ bps)

Peak *p* < 0.0387 (mouse)	91.3%	2.6%
Peak *p* < 0.0387 (dog)	90.7%	2.6%
*K*_*A*_/*K*_*S*_	90.5%	2.6%

**Table 3:** The distribution of RefSeq coding exons contained in the regions detected by the proposed method compared with those predicted by GENSCAN and TWINSCAN according to the types of exons: initial, internal, final, and single, where single refers to exons of single exon genes.

Exon type	Initial	Internal	Final	Single
Peak *p* < 0.0387 (mouse)	90.1%	94.3%	81.7%	80.1%
GENSCAN	81.8%	86.8%	78.3%	91.5%
TWINSCAN	30.4%	29.9%	42.4%	73.9%
